# Modification and Use of Naturally Renewable Cardanol-Type Prepolymers in One-Component Silyl-Terminated Prepolymer Sealant Systems

**DOI:** 10.3390/polym16243511

**Published:** 2024-12-17

**Authors:** Ritvars Berzins, Remo Merijs Meri, Janis Zicans, Agnese Abele, Volodymyr Sytar, Oleh Kabat, Anton Klymenko, Nataliia Mitina

**Affiliations:** 1 Institute of Polymer Materials, Faculty of Materials Science and Applied Chemistry, Riga Technical University, 3 Paula Valdena Street, LV-1048 Riga, Latvia; remo.merijs-meri@rtu.lv (R.M.M.); janis.zicans@rtu.lv (J.Z.); agnese.abele_1@rtu.lv (A.A.); 2 Department of Innovation Engineering, Ukrainian State University of Science and Technologies, 2 Lazariana Avenue, 49000 Dnipro, Ukraine; V.sytar@ua.fm (V.S.); Amber_UDHTU@i.ua (O.K.); natalimitina0000@gmail.com (N.M.); 3 Department of Software of Computer Systems, Dnipro University of Technology, 19 Dmytra Yavornytskoho Avenue, 49000 Dnipro, Ukraine; 03udhtu021990@ukr.net

**Keywords:** silyl-terminated, one-component sealant, cardanol

## Abstract

The current research is devoted to integrating naturally renewable cardanol derivatives into one-component silyl-terminated-polyether-based prepolymer systems to improve climatic resistance and obtain materials with versatile mechanical properties that could be significant to various sectors of the economy. Various cardanol-type products are used in industries that require high climatic resistance, and thus combining cardanol with commercially available silyl-terminated polyether prepolymers would improve its material climatic resistance, maintaining its market and application value as well as improving material sustainability. The results obtained in this work show that depending on how the cardanol prepolymer Ultra Lite 513 is modified, it is possible to increase the elasticity (670%) or tensile strength (104%) of the material as well as significantly increase the climatic resistance of the material, thus improving the quality and sustainability of the adhesive compared to existing silyl-terminated-prepolymer-based adhesives on the market.

## 1. Introduction

The use of raw materials that are renewable in nature is an important direction of research in practically all branches of chemistry, on which both a large part of the world’s scientific groups and industry representatives are working, slowly decreasing the proportion of use of oil-derived raw materials. Various raw materials can be found in nature that may be suitable for certain sectors of the economy, but in the current work, we focus on renewables that can be used to protect materials from climatic conditions: water and ultraviolet light. Today, various naturally occurring fatty acids, such as oleic acid, palmitic acid, stearic acid, castor oil, soybean oil, etc., are widely used individually or in compositions with various materials to endow desirable properties to certain end products [1,2]. Alkyl chains of fatty acids/oils typically increase the protection of the material against water and in some cases even improve the processing properties of the material by lubricating its surface [3,4]. In the current work, a cardanol-type molecule was chosen because it not only contains an alkyl chain, but also an aromatic ring with a hydroxyl group, which can be modified into various chemical compounds [5,6]. The alkyl group of the compound should increase the protection of the material against water, and the aromatic group should increase the absorption of ultraviolet light, endowing aging resistance. Another reason for choosing the compound was its availability and already widespread use in the coating and adhesive industry [7,8,9]. Cardanol is obtained as byproduct of the cashew nut industry, and its extraction quantity and application possibilities are increasing every year (in 2023, more than 900 kt), indicating that it is one of the most important raw materials obtained in nature [10,11]. The authors of the current manuscript took advantage of the properties of cardanol to improve the performance of a one-component silyl-terminated-polyether-type prepolymer system, which typically has relatively low climatic resistance. The modified cardanol compound is expected to form a combined prepolymer with the silyl-terminated polyether, reducing its hydrophilicity and making the material have more climatic resistance.

Silyl-terminated polyether prepolymers are widely used in different economic sectors, such as the construction industry, the automotive industry, and in metal and polymer constructions [12]. Silyl-terminated-polyether-type prepolymers are elastomers that have several advantages over polyurethane systems, such as no gas release during curing, no significant increase in viscosity due to decreasing temperature and greater safety for both the industrial operator and the customer [13].

In the current work, we used the commercially available epoxidized cardanol Ultra Lite 513, which was modified with two secondary amino silanes (Dynasylan 1189 and Dynasylan 1122), varying the functionality of the prepolymer. Silanes are the most widely used adhesion promoters in various polymer–metal–inorganic systems, especially in the adhesive and sealant industry [14,15,16]; however, they also work as polymer net cross-linkers, thus significantly influencing the mechanical properties of materials [17,18,19].

The results showed that Dynasylan 1189 mainly contributes to the formation of a linear polymer chain, while Dynasylan 1122 has six functional forms of material with higher tensile strength but loses its elasticity. The synthesized prepolymers can be integrated into a system by replacing up to 40% of the commercially available SAX 520, increasing the system’s climate stability while still maintaining adhesion to various substrates. The obtained cardanol-based prepolymers open the possibility of not only changing material mechanical, adhesion and rheological properties, but the full systems can be directly applied to adhesive/sealant materials that need higher climatic resistance, thereby increasing the systems’ overall application possibilities and in the process increasing material sustainability.

## 2. Materials and Methods

### 2.1. Materials

The materials used for the development of the prepolymer model systems and one-component sealants are summarized in Table 1.

### 2.2. Preparation of Model Systems and One-Component Sealant

Model systems were mixed using a SpeedMixer DAC 150 (mixer, Hauschild Speedmixer, Hamm, Germany) centrifugal laboratory mixer, casted in Teflon molds and cured at standard conditions—23 ± 2 °C, 50 ± 5% RH—for 1, 7 and 28 days.

The one-component sealant (full systems) was made by using a 3 L laboratory mixer, TEJA Engineering (Mixer, Teja Engineering Sp. z o.o. Zabkowice Slaskie, Poland). First, all liquid raw materials (prepolymers, compatibilizer/adhesion promoter and drying agent) were stirred for 5 min at 1000 rpm (central axis)/5 rpm (planetary axis); then, fillers were added through the reactor hatch, and the composition was stirred for 20 min at 3500 rpm (central axis)/35 rpm (planetary axis). Mixing was then continued under vacuum for 30 min at 3500 (central axis)/35 (planetary axis) rpm. The material was packed in 600 mL polypropylene cartridges.

### 2.3. Testing Methods

#### 2.3.1. Tensile Test

Tensile stress–strain measurements were carried out by using a Zwick/Roell Z010 universal testing machine (Mechanical properties testing machine, ZwickRoell GmbH & Co. KG, Ulm, Baden-Württemberg, Germany). The tests were performed according to ISO 527 at a test speed of 100 mm/min (dumbbell specimens). The testing was performed for the model and full systems after 1, 7 and 28 days of curing at 23 °C and 50% RH. The test result for each investigated system was reported as the average from five parallel measurements. Standard deviation was evaluated by using Microsoft Excel software.

#### 2.3.2. Rheology Tests

The viscosity of prepolymers was tested using the rheometer Bohlin CVO 100 (Rheometer, Malvern Instruments Ltd., Worcs, UK). The instrument was equipped with a 20 mm diameter spindle with plate–plate geometry (gap size 1000 µm). The tests were conducted at different shear rates at a constant temperature of 25 °C.

#### 2.3.3. Aging of Materials in a Climatic Chamber

The one-component model and full systems were aged in a climatic chamber (Climatic chamber, Q-Lab Corporation, Bolton, UK) with the following aging conditions: 55 °C, 95% humidity and UV (UVA: 340+ lamps irradiating the material in the region of 365 to 295 nm were used for irradiation). The systems were aged for 252 and 504 h, relaxed at standard conditions (23 ± 2 °C, 50 ± 5% RH) and subjected to tensile mechanical analysis.

#### 2.3.4. Lap Shear Strength Test

This method consists of determining the magnitude of the shear destructive capability during stretching of a standard sample with efforts that try to shift one-half of the sample relative to the other one.

**Figure 1:**
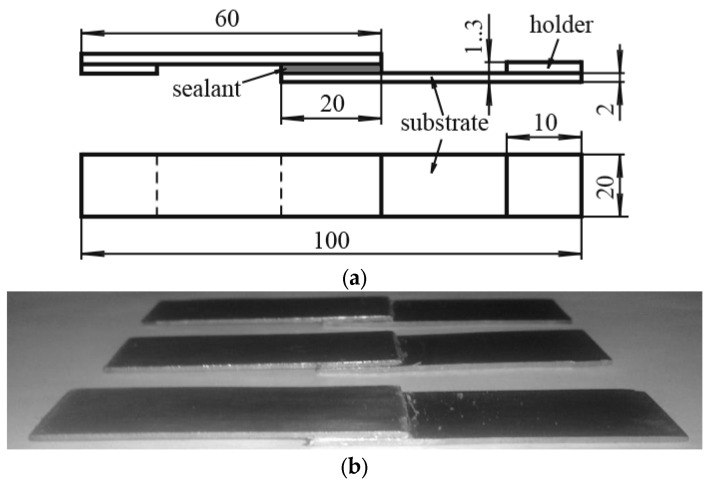
Schematic (**a**) and photographic (**b**) sample for quantitative determination of shear strength of adhesion joint.

The lap shear strength of the adhesive joint was determined with the tensile testing machine 2166 P-5 with the measurement accuracy of efforts up to 1%. In order to determine the shear adhesive joint strength, the samples were obtained in accordance with ASTM D3164M. The sample is the two metal plates connected in an overlapping way by means of a 5 mm thick layer of polymer sealant, which thus acts as an intermediate element of the substrate–sealant–substrate system (Figure 1).

#### 2.3.5. Synthesis of a Silyl-Terminated Prepolymer Using Modified Cardanol Ultra Lite 513

##### Synthesis of SIL 1189 Prepolymer

An amount of 100 g of Ultra Lite 513 was weighed into a 500 mL flask, to which 70 g of Dynasylan 1189 was added. The mixture was stirred for 2 h at 60 °C under nitrogen, forming an orange liquid with a viscosity of 0.23 Pa·s. The prepolymer was used without purification, yielding 167 g of material (98.2%). The synthesis process was characterized by infrared spectroscopy, FTIR: 2925 (C-H), 2854 (C-H), 1583 (C=C), 1485 (C=C), 1447 (C=C), 1260 (C-O), 1039 (Si-O), 909 (C-H) and 814 (C-H) cm^−1^.

##### Synthesis of SIL 1122 Prepolymer

An amount of 100 g of Ultra Lite 513 was weighed into a 500 mL flask, to which 106 g of Dynasylan 1122 was added. The mixture was stirred for 2 h at 60 °C under nitrogen, forming an orange/brown liquid with a viscosity of 0.51 Pa·s. The prepolymer was used without purification, yielding 203.2 g of material (98.6%). The synthesis process was characterized by infrared spectroscopy, FTIR: 2925 (C-H), 2855 (C-H), 1584 (C=C), 1444 (C=C), 1261 (C-O), 1035 (Si-O), 995 (C-H), 949 (C-H) and 879 (C-H) cm^−1^.

## 3. Results

### 3.1. Mechanical (Tensile) Properties of SAX 520/SIL 1189 and SIL 1122 Systems

#### 3.1.1. Mechanical (Tensile) and Aging Properties of SAX 520/SIL 1189 and SIL 1122 Model Systems

**Figure 2:**
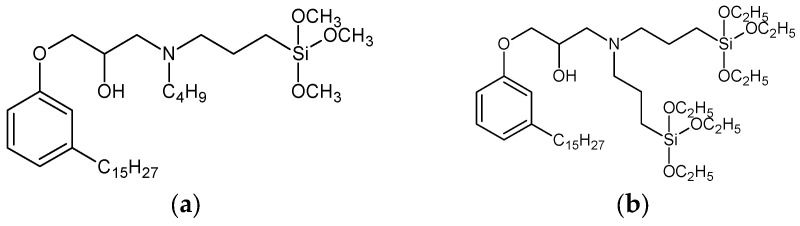
Synthesized silyl-terminated prepolymers SIL 1189 (**a**) and SIL 1122 (**b**).

The model system consisted of prepolymers, 10% of filler and a catalyst (Table 2). The commercially available SAX 520 was used as the base prepolymer. The concentration of the synthesized prepolymer SIL 1189 (Figure 2a) or SIL 1122 (Figure 2b) was increased until no improvement in the mechanical properties of the system was observed.

**Figure 3:**
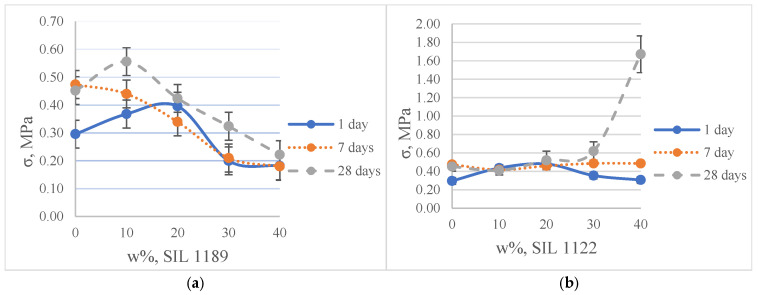
Tensile strength of model systems with commercial SAX 520 and self-synthesized SIL 1189 (**a**) or SIL 1122 (**b**) silyl-terminated prepolymers.

The self-synthesized prepolymers SIL 1189 and SIL 1122 had significant impacts on the SAX 520 tensile properties: one of them acts as a polymer network linearizer, reducing the tensile strength of the material (SIL 1189), and the other as a system cross-linker (SIL 1122), increasing the tensile strength (Table 2). This can be explained by higher material functionality, which will lead to more cross-linked material. For the system with SIL 1189 (Figure 3a), a decrease in tensile strength was observed at 20% of prepolymer SIL 1189, but at 40%, it had already decreased by 104% (0.23 MPa), indicating that the polymer net forms a more linear structure. For the system with SIL 1122 (Figure 3b), the tensile strength at a 40% prepolymer concentration increased by 271% after curing for 28 days at 23 °C and 50% RH. A significant effect of SIL 1122 only appeared at a relatively high concentration because the SAX 520 prepolymer is relatively linear and the structure needs a high concentration of cross-linker to increase the material’s tensile strength.

**Figure 4:**
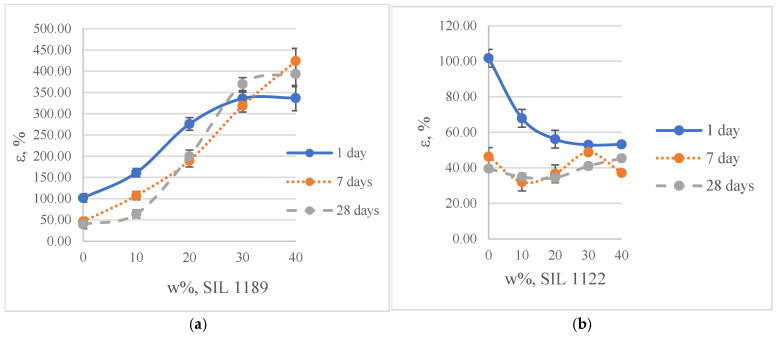
Tensile deformation of model systems with commercial SAX 520 and self-synthesized SIL 1189 (**a**) or SIL 1122 (**b**) silyl-terminated prepolymers.

When SIL 1189 (Figure 4a) was used at a 10% prepolymer content, the total deformation value of the polymer composition increased by more than 50% after 1 day, indicating that the prepolymer (SIL 1189) is an effective linearization additive. Increasing the prepolymer to 40% increased the material deformation value by 227% after 1 day of curing at 50% RH. When the system was subjected to 28 days of curing at 23 °C and 50% RH, the deformation values of the systems decreased when the SIL 1189 concentration was 10% or lower, but upon increasing it further, the deformation also increased, implying that the prepolymer does not cross-link over time but only increases its molecular weight. The concentration of SIL 1189 was not further increased, because at a concentration of w_SIL1189_ = 40%, the surface of the material was too sticky and could not be used in real applications. The deformation values of the polymer composition decreased with the addition of the prepolymer SIL 1122 (Figure 4b), but the largest decrease was with the addition of 10%; a further increase did not result in a significant decrease in deformation after 1 day. When the materials were further cured at 23 °C and 50% RH (7 and 28 days), the values of all polymer concentrations practically leveled off, forming deformations around 40%. The prepolymer concentration of SIL 1122 was not increased to make the results more comparable to the effect of SIL 1189 on the system properties.

**Figure 5:**
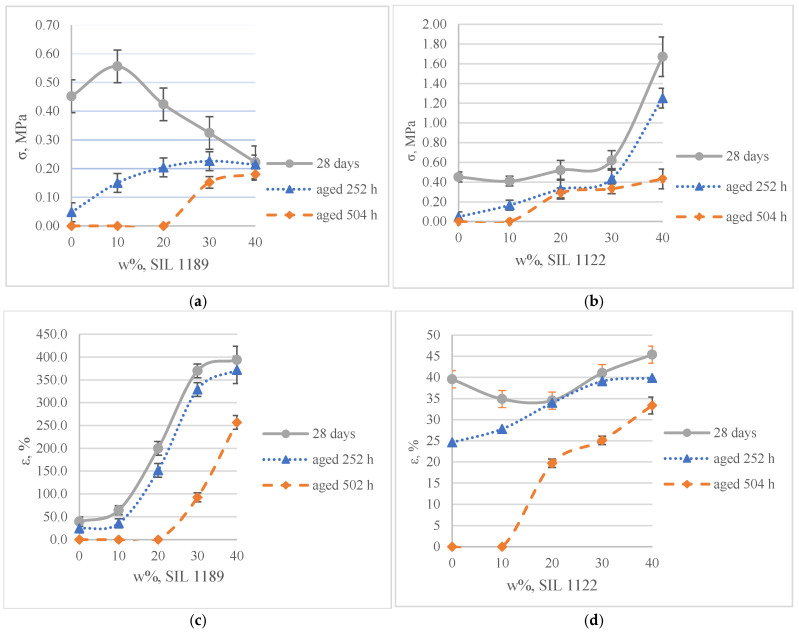
Mechanical properties of systems with SIL 1189 (**a**,**c**) and SIL 1122 (**b**,**d**) regarding tension after aging of materials for 252 and 504 h in a climatic chamber.

It has already been described in the literature that cardanol-type compounds are used in various applications that require protection against various climatic conditions [7,8,9]. The alkyl chains in the cardanol molecule are an effective hydrophobic element that repels water molecules, and the aromatic ring is able to absorb part of the ultraviolet light radiation. The results showed that increasing concentrations of both synthesized prepolymers SIL 1189 and SIL 1122 improve the material’s resistance to climatic aging. The SIL 1122 (Figure 5b,d) prepolymer already shows a significant improvement in the climatic stability of the material at 20%; compared to SIL 1189 (Figure 5a,c), this kind improvement is at 30%, which can be explained by the fact that SIL 1122 forms a more cross-linked system in which it is more difficult to influence climatic conditions. The studied systems show that the synthesized prepolymers are not only able to influence the mechanical properties of the material but also improve its resistance to combined water, UV light and temperature impacts.

Analyzing the results of the model systems, it can be seen that the two obtained prepolymers (SIL 1189 and SIL 1122) were able to significantly change the mechanical properties of the commercial material and increase the protection against climatic conditions. It should be emphasized that an important aspect of the work is that renewable raw materials were used, which makes this system more sustainable.

In the next stage of work, full adhesive systems were developed. They were developed to show how the material will compare to real systems that are sold in the adhesive and sealant market.

#### 3.1.2. Tensile, Rheological and Adhesive Properties of Full Systems

Table 3 shows the composition of the one-component full systems that were packaged in aluminum cartridges and used to determine the mechanical, rheological, adhesive and aging properties.

Typically, adhesive and sealant systems consist of polymers, fillers, adhesion promoters and catalyst systems (Table 3). The systems here are comparable to the materials in the adhesive/sealant market. The developed one-component systems could not be tested after 1 day, as many of the recipes were not completely solidified throughout the volume, which is typical for such systems, and it is mostly not necessary to ensure the transport of the material after 1 day, so the mechanical properties were measured regarding tension after 7 and 28 days of curing at 23 °C and 50% RH.

After curing the materials for 7 days at 23 °C and 50% RH, SIL 1189 and SIL 1122 at their maximum tensile values increased the tensile strength of the materials by 0.4 and 0.45 MPa, respectively, indicating that the synthesized prepolymers have not fully reacted and affected the properties of the polymer even after 7 days. After 28 days of curing, the systems with SIL 1189 significantly increased the tensile strength with the addition of 20% of the prepolymer but reached their maximum at 30%, which was 84% higher than the system with the neat SAX 520 prepolymer. By substituting 40% of the SAX 520 prepolymer, the material became too soft and tacky, and because of that we did not increase its concentration even more. SIL 1189 linearized the polymer network of the material, and at a certain concentration, the cross-linking of the material decreased so far that the material became tacky. The systems with SIL 1122 significantly increased the tensile strength only by substituting 40% of the SAX 520 prepolymer; the increase was 104% (compared to the neat SAX 520 system), and the surface of these materials remained less tacky when the concentration of the synthesized prepolymer was increased. The results indicate that it would be possible to further increase the concentration of the SIL 1122 prepolymer to even higher values, but we chose not to increase it further, because since the prepolymer concentration was not increased for the system in which SIL 1189 was integrated, we chose not to take this step for systems with SIL 1122 either.

**Figure 6:**
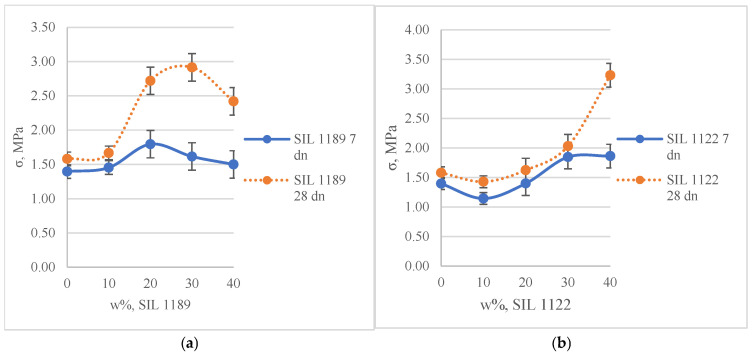
Tensile strength of full systems with commercial SAX 520 and self-synthesized SIL 1189 (**a**) or SIL 1122 (**b**) silyl-terminated prepolymers.

**Figure 7:**
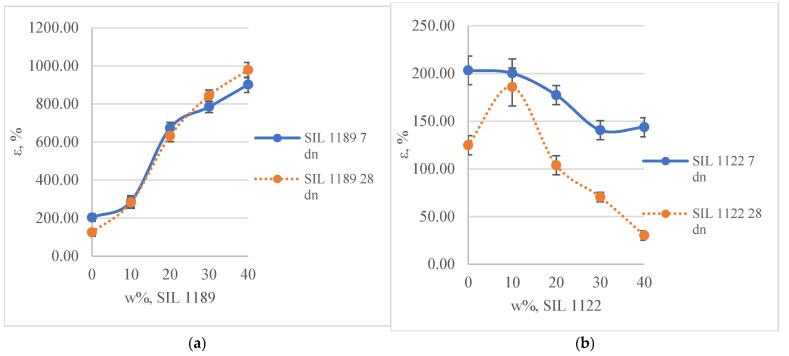
Tensile deformation of full systems with commercial SAX 520 and self-synthesized SIL 1189 (**a**) or SIL 1122 (**b**) silyl-terminated prepolymers.

Integrating the SIL 1189 prepolymer (Figure 6a and Figure 7a) into the systems increased the tensile deformation of the material, and by replacing 40% of the SAX 520 prepolymer, it increased by 688%. However, the values practically did not change from 7 to 28 days, which can be explained by the fact that the molecular weight of the polymer increases but the cross-link density does not change, as a result of which the tensile strength of the material increases but the deformation properties do not change. When SIL 1122 (Figure 6b and Figure 7b) is integrated into the system, the tensile deformation properties of the material decrease due to the increase in cross-link density. After 7 days, the reduction in deformation was still less pronounced, but after 28 days at 40% SIL 1122 it was 534% of the system with only SAX 520, which can be explained by the fact that it takes more time for the system to fully cross-link.

The developed full adhesive systems show high mechanical properties, which make them very competitive in the adhesives and sealant market. Especially good results were shown by the systems that integrated the prepolymer SIL 1189, which not only increased the tensile strength of the material but also the deformation.

#### 3.1.3. Full-System Rheological Properties

The viscosity of the prepolymers µ is as follows: μ_SAX 520_ = 46 Pa*s, μ_SIL1189_ = 0.23 Pa*s and μ_SIL1122_ = 0.51 Pa*s.

**Figure 8:**
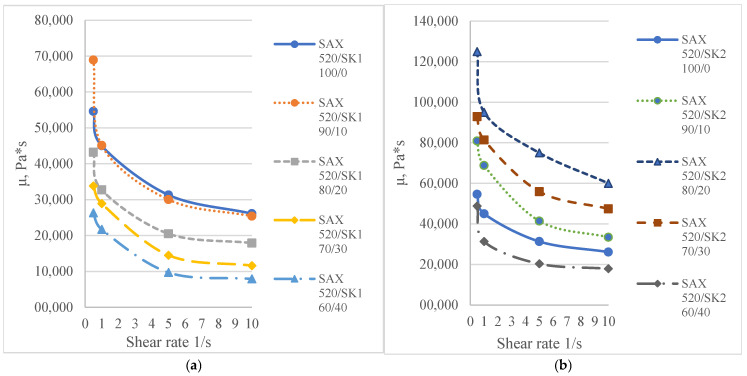
System with SIL 1189 (**a**) and SIL 1122 (**b**) viscosity changes as a function of shear rate.

When replacing SAX 520 with the SIL 1189 prepolymer system, the viscosity of the material decreases (Table 4). A significant reduction was observed when adding at least 20% of the prepolymer (~30%). By increasing the content of the SIL 1189 (Figure 8a) prepolymer to 40%, the viscosity of the material at a high shear rate (10) decreased from 26,300 to 5100 Pa*s (416%). The viscosity of the material decreased due to the much lower viscosity of the SIL 1189 prepolymer compared to the SAX 520 prepolymer. The decrease in viscosity allows an increase in the content of fillers in the material, which potentially reduces the cost of the material while still maintaining its workability. At a low concentration of SIL 1189, the viscosity of the material increased, which typically occurs because the added material concentration is too low and does not form a homogeneous material throughout the volume. When replacing SAX 520 with the SIL 1122 prepolymer (Figure 8b), there was a significant reduction in viscosity only when the prepolymer concentration reached 40% of the total prepolymer concentration. The SIL 1122 prepolymer not only has higher functionality but also has a bigger source of hydrogen bonds that can form physical bonds with other elements in the material, which increases the material viscosity at a lower concentration of SIL 1122; however, as its concentration increases, the cooperation of hydrogen bonds becomes weaker, resulting in the reduction of material viscosity because of the lower viscosity of SIL 1122 compared to SAX 520.

Synthesized prepolymers reduce the viscosity of the overall system, thus allowing more fillers to be added to the system, which will reduce the price of the material, making the resulting system more attractive from a commercial point of view.

#### 3.1.4. Lap Shear Strength of Adhesive Joint of SAX 520/SIL 1189 and SIL 1122 Full Systems

One of the main indicators for sealing materials is their adhesive properties to substrates of various natures.

Therefore, the aim of this work is the detailed experimental study of the obtained prepolymers based on SIL 1189 and SIL 1122 on the adhesive properties when added to the commercially available SAX 520 material.

**Figure 9:**
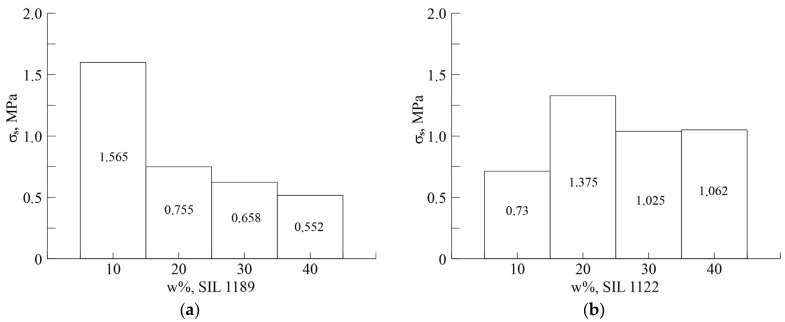
The shear strength (σ_s_) of the adhesive joint (sealant–metal substrate) depending on the content of prepolymers based on SIL 1189 (**a**) and SIL 1122 (**b**) in the commercially available SAX 520 material.

Figure 9 shows the results of the experiments carried out to determine the shear strength of the adhesive joint on metal substrates.

According to this research, it can be concluded that the shear strength of the adhesive joint with metal substrates and the sealant varies between 0.500 and 1.565 MPa depending on the type of prepolymer (SIL 1189 or SIL 1122) and its concentration in SAX 520. The highest lap shear values were observed in systems with SIL 1189 at a 10% prepolymer content; however, SIL 1122 was used at 20%. The values of these parameters for the studied systems are 1.565 and 1.375 MPa, respectively. These values are at the level of or exceed the best commercial sealant analogues (for example, the shear strength of the SOUDASEAL 240FC sealant–metal substrate bond is 1.39 MPa, and for the Tenalux^®^ 131 sealant, it is 0.99 MPa).

**Figure 10:**
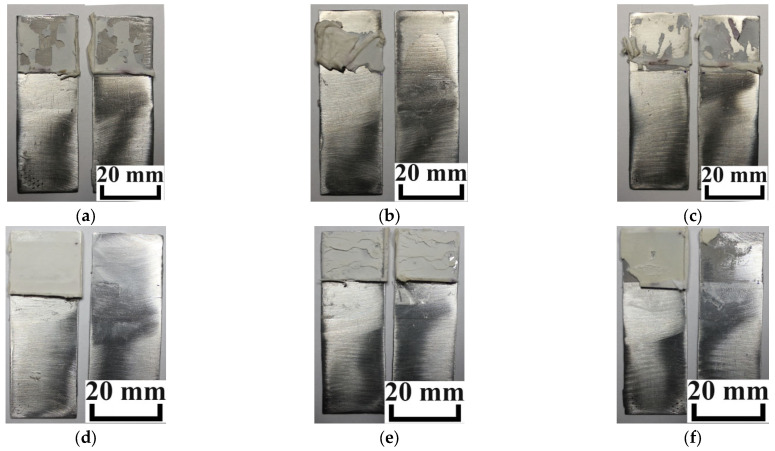
Photos of the nature of fracture of the studied adhesion joint of the samples with SIL 1189 prepolymer (**a**–**c**) and SIL 1122 (**d**–**f**) in SAX 520 with different prepolymer contents (%): (**a**–**d**) 10%; (**b**–**e**) 20%; (**c**–**f**) 30%.

It is also of interest to study the nature of fracture of the studied adhesion joint. Figure 10 shows photos of the samples after the experiments.

The received data show that the adhesive joints with the highest shear strengths are mainly destroyed cohesively (Figure 10a,e). This is due to their high level of interaction with the metal substrates. For less strong bonds, adhesive destruction is more typical (Figure 10b–d,f). This indicates that the optimal composition of the developed sealants has been found (for the SAX 520/SIL 1189 system, this is 10% prepolymer; for the SAX 520/SIL 1122 system, 20%) in terms of their adhesive strength to metal substrates.

## 4. Conclusions

(1) The test results for the model systems showed that by modifying the Ultra Lite 513 molecule with the silane Dynasilan 1189, it is possible to make the polymer structure more elastic, but by adding Dynasylan 1122, it is possible to increase the tensile strength of the material. It is possible to increase the elasticity of the material by 894% by adding 40% of SIL 1189 to the commercial prepolymer SAX 520 after 28 days at 23 °C and 50% RH, but even at this concentration the material becomes too soft and sticky. By adding SIL 1122 to the material, it is possible to increase the tensile strength of the material by 171% by replacing 40% of the SAX 520 prepolymer after 28 days at 23 °C and 50% RH.
(2) Both modified prepolymers (SIL 1189 and SIL 1122) increased the climatic resistance of the material, and increasing their concentration improves the climatic resistance. As the concentration of the modified prepolymers increased, the deviation of the mechanical properties from the initial tensile strength decreased, indicating that the modified prepolymers protect the overall model system.
(3) In the developed full adhesive system, the tensile strength properties increased both for systems without and with the prepolymers SIL 1189 and 1122. The compositions with the prepolymer SIL 1189 reached their maximum tensile strength at a 30% polymer concentration (2.92 MPa); at this concentration, they also had high deformation (843%). When the SIL 1122 prepolymer was added to the composition, the increase in adhesive tensile strength was higher (3.23 MPa at SIL 1122 40%) compared to SIL 1189, but at higher concentrations, a decrease in deformation (30.2% at SIL 1122 40%) was also observed, indicating that the prepolymer acted as a cross-linker of the system.
(4) The lap shear strength of the adhesive joint of the prepolymers based on SIL 1189 and SIL 1122 in the commercially available SAX 520 material was determined. It was established that the highest values of this parameter (1.565 and 1.375 MPa) are for the SAX 520/SIL 1189 system with 10% prepolymer (1) and the SAX 520/SIL 1122 system with 20%. It was established that the strongest adhesive joints are mainly destroyed cohesively due to their high level of interaction with metal substrates.
(5) The synthesized prepolymers SIL 1189 and SIL 1122 have approximately 100 times lower viscosity compared to the commercially available SAX 520, which means that when integrating the synthesized prepolymers into the system, the viscosity of the adhesives decreases, which potentially allows increasing the content of fillers in the material, thus potentially reducing the cost of the adhesive.
(6) The developed compositions are competitive with commercially available adhesives and also significantly improve the mechanical, rheological and climatic properties of the typical commercially available prepolymer SAX 520.
(7) Synthesized prepolymers of the cardanol type are more expensive than commercial products, but their effect, even at low prepolymer concentrations (20–40% of the prepolymer composition), on the material’s mechanical properties and climatic resistance is very significant. The effect of the synthesized cardanol-based prepolymers makes them a different class of material, with a higher price than typical sealants and adhesives used in construction. This type of material could potentially be used as a structural adhesive or in the automotive industry. In these industries, the material added value is higher; therefore, despite the price increase caused by the synthesized cardanol prepolymer, the profit obtained will be higher.

## Figures and Tables

**Table 1:** Components of the model systems and one-component full-sealant system.

Raw Material Class	Trade Name	Raw Material Structure Formula
Prepolymer	SAX 520 (Kaneka, Westerlo-Oevel, Belgium)	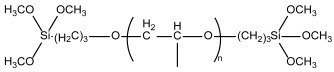
Prepolymer	Ultra Lite 513 (Cardolite, Gent, Belgium)	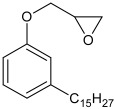
Silane	Dynasylan 1189 (Evonik, Marl, Germany)	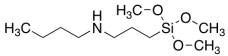
Silane	Dynasylan 1122 (Evonik, Marl, Germany)	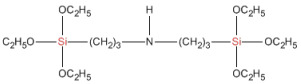
Drying agent	Dynasylan VTMO (Evonik, Marl, Germany)	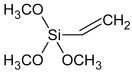
Adhesion promoter	Dynasylan Glymo (Evonik, Marl, Germany)	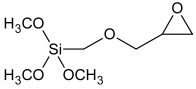
Filler	Omycarb 1TVA (Omya, Oftringen, Switzerland)	Coated natural calcium carbonate
Filler	Hakuenka CCR-S10 (Omya, Oftringen, Switzerland)	Coated precipitated calcium carbonate
Catalyst	Tibcat 318 (TIB chemicals, Mannheim, Germany)	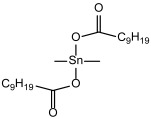

**Table 2:** Model system mixtures.

Raw Materials	Mass, g				
SAX 520	90	81	72	63	54
Synthesized SIL prepolymer (SIL 1189 or 1122)	0	9	18	27	36
Hakuenka CCR-S10	10	10	10	10	10
Tibcat 318	0.4	0.4	0.4	0.4	0.4

**Table 3:** Full one-component silyl-terminated prepolymer systems.

Raw Materials	Mass, g			
SAX 520	36	32	28	24
SIL 1189/1122	4	8	12	16
Dynasylan VTMO	0.8	0.8	0.8	0.8
Dynasylan Glymo	0.2	0.2	0.2	0.2
Omycarb 1TVA	10	10	10	10
Hakuenka CCR-S10	43.6	43.6	43.6	43.6
Tibcat 318	0.4	0.4	0.4	0.4

**Table 4:** Full-system viscosity changes as a function of shear rate.

		SK1 (SIL 1189)				SK2 (SIL 1122)			
	100_0	90_10	80_20	70_30	60_40	90_10	80_20	70_30	60_40
Shear rate (1/s)	μ, Pa*s								
0.5	54,600	68,900	43,200	33,800	26,300	80,900	12,5000	92,900	48,500
1	45,300	45,100	32,700	28,900	21,700	68,800	95,000	81,400	31,300
5	31,300	30,000	20,500	14,500	9700	41,500	75,000	55,900	20,400
10	26,100	25,400	17,900	11,600	7900	33,100	59,100	47,800	17,900

## Data Availability

The data presented in this study are available on request from the corresponding author.
